# Attachment style and its impact on connection to God in individuals with brain injury: behavioral and lesion-based findings

**DOI:** 10.3389/fneur.2025.1488890

**Published:** 2025-04-24

**Authors:** Shira Cohen-Zimerman, Irene Cristofori, Patrick McNamara, Frank Krueger, Barry Gordon, Jordan Grafman

**Affiliations:** ^1^Cognitive Neuroscience Laboratory, Brain Injury Research, Shirley Ryan AbilityLab, Chicago, IL, United States; ^2^Department of Physical Medicine and Rehabilitation, Feinberg School of Medicine, Northwestern University, Chicago, IL, United States; ^3^Institute of Cognitive Sciences Marc Jeannerod, CNRS/UMR 5229, Bron, France; ^4^University Claude Bernard Lyon 1, Villeurbanne, France; ^5^Department of Psychology, National University, San Diego, CA, United States; ^6^Department of Neurology, Boston University, Boston, MA, United States; ^7^School of Systems Biology, George Mason University, Fairfax, VA, United States; ^8^Department of Psychology, University of Mannheim, Mannheim, Germany; ^9^Department of Neurology, Johns Hopkins University School of Medicine, Baltimore, MD, United States; ^10^Department of Cognitive Science, Johns Hopkins University, Baltimore, MD, United States; ^11^Departments of Neurology, Northwestern University, Chicago, IL, United States; ^12^Departments of Psychiatry, Northwestern University, Chicago, IL, United States; ^13^Cognitive Neurology & Alzheimer's Disease, Feinberg School of Medicine, Northwestern University, Chicago, IL, United States; ^14^Department of Psychology, Northwestern University, Chicago, IL, United States

**Keywords:** attachment style, connection to God, voxel-based lesion-symptom mapping (VLSM), traumatic brain injury (TBI), orbitofrontal cortex (OFC)

## Abstract

Attachment style shapes one's connections with important figures in their life. One such unique relationship is the connection to God (CTG), which may be shaped by attachment style. Stronger CTG has been associated with secure attachment, yet the neural mechanisms underlying this relationship remain unclear. While previous research has implicated the prefrontal cortex (PFC) in CTG, findings have been mixed and may depend on attachment style—an idea that has yet to be directly tested. This study aimed to (1) examine whether individuals with a secure attachment style report higher levels of CTG compared to those with a non-secure attachment style, and (2) identify the brain regions associated with CTG in individuals with secure vs. non-secure attachment. We assessed attachment style and CTG in a sample of male combat veterans (*N* = 150), the majority of whom had focal traumatic brain injuries (pTBI; *N* = 119). Brain imaging (CT scans) was also obtained. Behaviorally, after controlling for age, years of education, and brain volume loss, individuals with a secure attachment style reported stronger CTG. Voxel-based lesion-symptom mapping revealed that damage to the right orbitofrontal cortex was associated with stronger CTG in individuals with secure—but not insecure—attachment. These findings suggest that attachment style shapes CTG at both behavioral and neural levels. Moreover, they highlight the potential role of attachment style in TBI recovery, offering insights that could inform spiritually integrated therapeutic interventions and support strategies.

## 1 Introduction

The desire to form meaningful relationships with others is a fundamental human need. Attachment theory (1) is a psychological framework that suggests that early relationships with caregivers shape an individual's emotional bonds and how they relate to others throughout adult life (2, 3). Adult attachment is classified into four styles: a *secure* style and three *insecure* subtypes—*fearful, preoccupied*, and *dismissive*. A large body of research suggests that attachment style can impact one's self-esteem, romantic relationships and friendships (4–6).

Just as attachment patterns influence interpersonal relationships, they can also extend to one's connection with God, influencing the perception of God as a secure or insecure attachment figure (7–9). Specifically, people with secure attachment styles were shown to be more likely to develop a secure, positive relationship with God, viewing God as a supportive and loving figure (10, 11).

Traumatic brain injury (TBI) is a major global health challenge with profound impacts on survivors and their families (12). Despite its widespread effects, there is limited knowledge on how attachment style influences the recovery journey. Specifically, the impact of attachment style on an individual's connection to God after suffering a TBI, and the neuronal mechanisms underlying such impact, remain unexplored.

### 1.1 Neural basis of connection to God

Religious belief and connection to God are uniquely human experiences, yet their neural basis was unexamined for years. However, over the past two decades, a growing number of studies have aimed to uncover the neuronal underpinnings of these experiences (13, 14). For instance, regions within the social cognition network—such as the temporopolar region, medial prefrontal cortex, temporoparietal junction, and precuneus—were shown to be active during personal prayers to God (15). This aligns with the idea that religious individuals often perceive God as a relational partner in their religious practices.

In particular, the prefrontal cortex (PFC) has been suggested as a key region associated with religious belief and connection to God. This is consistent with the PFC's role in social relationships, affective processing, and its connections to the reward network. However, while some studies found that the PFC plays a role in religiosity and the experience of connection to God (16, 17), others have reported no significant involvement of this region (18, 19).

In summary, the current literature presents mixed findings regarding the PFC's role in one's connection to God (20, 21). While some studies suggest its involvement, it remains unclear whether this relationship is consistent or influenced by individual differences. One possibility is that the PFC's role on connection to God depends on attachment style, but this hypothesis has yet to be directly tested. However, although attachment theory has generated a rich body of research, the neural bases of its potential impact remain unknown. Investigating this intersection could provide valuable insights into the role of attachment in the spiritual aspects of recovery, potentially informing targeted therapeutic interventions.

### 1.2 Current study

The primary objective of this study is to investigate how attachment style can impact one's connection to God on the behavioral and neuronal level. The sample included 150 combat veterans who participated in the Vietnam Head Injury Study (VHIS) (22, 23). The majority of this sample had localized penetrating traumatic brain injuries (*N* = 119).

To examine impact of attachment style on connection to God on the behavioral level, we collected data from two surveys assessing attachment style [i.e., the relationship questionnaire (RQ) (24), and the Relationship Scales Questionnaire (RSQ) (25)], as well as three surveys assessing connection to God [i.e., selected items from the God Image Inventory (26); the religious experience scale (27); and the religious emphasis scale (28), see detailed description below].

To examine the underlying neuronal involvement, we conducted a Voxel-Based Lesion Symptom Mapping (VLSM) on individuals with secure and insecure attachment style. This technique analyzes voxel-wise brain damage across individuals to identify brain regions associated with a behavioral outcome.

This an exploratory study with two aims. The first aim of this study was to examine whether individuals with a secure attachment style demonstrate elevated levels of connection to God compared to individuals with a non-secure attachment style, regardless of brain damage. The second aim was to identify, using VLSM, the brain areas associated with connection to God in individuals with secure attachment, relative to individuals with non-secure attachment.

## 2 Materials and methods

### 2.1 Participants

Participants were drawn from phase IV of the W.F. Caveness Vietnam Head Injury Study (VHIS) registry, which is a prospective, long-term follow-up observational study of male veterans with focal penetrating traumatic brain injury (pTBI) and with no brain injury (no-BI). During phase four of the study (2008–2012; ~40–45 years post-injury) we assessed 150 individuals, 119 of them with pTBI and 31 with no brain injury. The age range of participants was 59–81 years, with 98% (146/150) being 69 years or younger. The pTBI and no-BI groups were matched with respect to age, level of education, and pre-injury general intelligence, measured using the Armed Forces Qualification Test (AFQT; Table 1).

**Table 1:** Demographic characteristics and neuropsychological tests scores across groups.

**Group demographics**	**Group**	***N***	**Mean**	**SD**	***P***	**Cohen's d**
Age (years)	pTBI	119	63.387	2.94	0.499	0.137
	No-BI	31	62.968	3.507		
Education (years)	pTBI	119	14.639	2.235	0.276	−0.221
	No-BI	31	15.129	2.172		
Pre-injury AFQT	pTBI	107	65.84	23.26	0.288	−0.254
	No-BI	21	71.57	18.08		
Post-injury AFQT	pTBI	116	57.086	25.376	0.002	−0.652
	No-BI	30	72.967	19.779		

Table presents mean, SD, p-values and effect size (Cohen's d) for penetrating traumatic brain injury (pTBI) patients and no brain injury (no-BI) controls.

The AFQT represents the Armed Forces Qualification Test, a classification measure highly correlated with intellectual ability.

**Figure 1:**
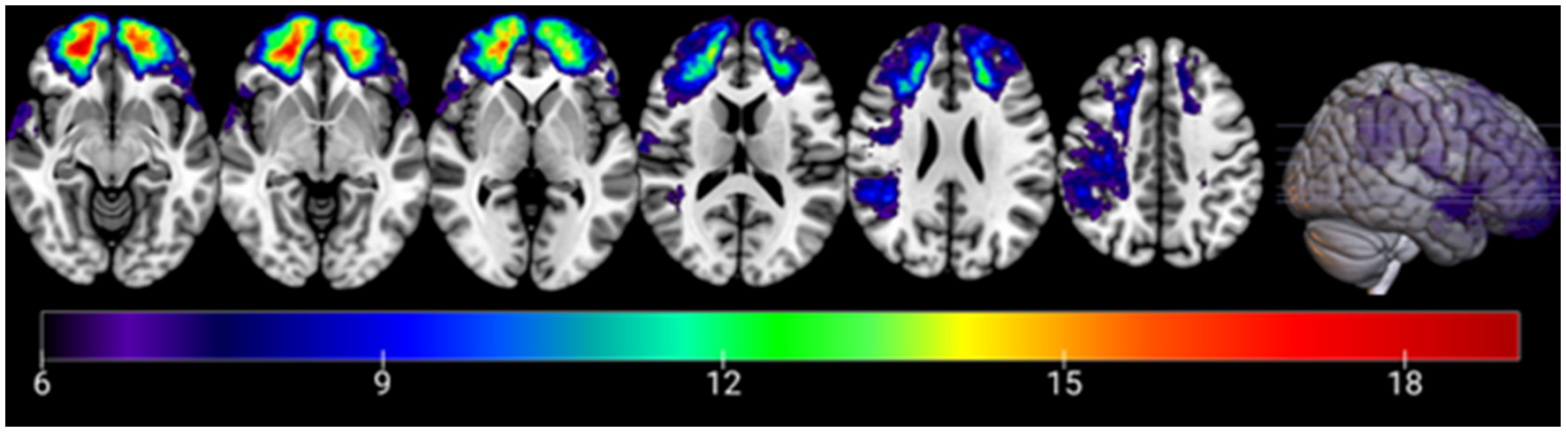
Overlay density map of the lesions in the penetrating traumatic brain injury group. Numbers on the scale represent the number of patients with lesion in a specific voxel (6 patients threshold). The color legend indicates the number of patients with damage to a particular voxel. *Z* values from left to right: −10, −5, 0, 15, 25, 40. Images are in radiological space (i.e., right is left).

As one would expect, the pTBI group scored lower than controls on a measure of post-injury cognitive abilities (AFQT) (29). However, both groups scored within the normal range of the test scores (above the 50th percentile). The lesion overlay density map for all participants in the pTBI group can be found in Figure 1.

The study was approved by an Institutional Review Board at the National Institute of Neurological Disorders and Stroke at the National Institute of Health, Bethesda, MD, USA, and the current analysis was approved by the Northwestern University Institutional Review Board.

### 2.2 CT acquisition and analysis

Axial CT scans without contrast were obtained using a GE Medical Systems Light Speed Plus CT scanner at the Bethesda Naval Hospital, Bethesda, MD. These scans were conducted during Phase 3 of the Vietnam Head Injury Study (2003–2006). Although more recent CT scans were conducted during Phase 4 (2008–2012) for clinical purposes, an NIH staff radiologist reviewed them and reported no new lesions or significant pathological changes compared to Phase 3. CT scans were utilized because most of the pTBI participants had retained metal in their brain.

Structural neuroimaging data were reconstructed with an in-plane voxel size of 0.4 x 0.4 mm, an overlapping slice thickness of 2.5 mm, and a 1-mm slice interval. Lesion location and volume from CT images were determined using the interactive ABLe software (30), implemented in MEDx v3.44 (Medical Numerics) with enhancements to support the Automated Anatomical Labeling (AAL) atlas (31).

Each scan was normalized to a CT template brain image in Montreal Neurological Institute (MNI) space. A trained neuropsychiatrist performed manual tracing for each lesion, which was later reviewed by a blinded observer (J.G.) to reach a consensus on lesion extent.

### 2.3 Voxel-based lesion-symptom mapping

A VLSM analysis (32) was applied to test the association between the traced brain lesions for each participant and their responses on the surveys measuring CTG. In this VLSM analysis, the CTG scores of patients with a lesion in each voxel are compared to CTG score of patients without a lesion in this voxel using a *t*-test (see detailed description of CTG measures below). To have sufficient statistical power and to be able to test regions all over the brain, voxels that did not have at least 6 patients with damage were excluded from the analysis. Note that the overlay map of lesion locations for 119 patients (Figure 1) map shows brain regions with lesions present for at least six participants in each voxel consistent with the constraints of the VLSM as described above. The map shows a sufficient degree of overlap to draw conclusions for all the target brain regions. Participants' lesion size was used as a covariate in this analysis.

The analysis was carried out using the VLSM package version 2.60 (https://aphasialab.org/vlsm/) on MATLAB R2022a (Mathworks, Natick, MA) software. Anatomic labeling was performed with Automated Anatomical Labeling (AAL) atlas, and Natbrainlab atlas of white matter pathways (33). VLSM results were visualized in MRICronGL (https://www.nitrc.org/projects/mricrogl) overlaid on an MNI standard brain. All *t*-tests were two-tailed and a *P* < 0.05 was considered statistically significant.

### 2.4 Neuropsychological testing

Participants were assessed from 2009 to 2012 at the National Institutes of Health in Bethesda, MD, over a 5- to 7-day period with tests that measured a wide variety of cognitive, social and personality measures. For this study, we focused on the assessment of connection to God and attachment style.

#### 2.4.1 Connection to God

Participants completed the following 3 scales:

1. *God Image Inventory* (26). This inventory reflects an internal model of the sort of person that the individual imagines God to be. The original God Image Inventory consists of 156 items. Since our participants were assessed in a variety of tasks and questionnaires over a week, we decided to select 14 items from the *Presence and Salience subscales* of the God Image Inventory. The Cronbach's alpha for the God Image Inventory is 0.958. Examples of the items are: “*I can talk to God on an intimate basis,”* “*God tells me what he wants from me.”* A higher score indicates a stronger sense of attachment to God and the presence of God. See Supplementary Text S1 for the complete list of God Image Inventory items we used.
2. Religious *experience scale* (27). The scale reflects the perceived influence of God in one's life, including feelings of being forgiven for sins and referring to God when making decisions. This scale consists of 12 items. The Cronbach's alpha for the Religious Experience Scale is 0.96. Examples of items are “*I experience an awareness of God's love*” and “*I pray privately in places other than church.”* A higher score indicates a stronger influence of God in the individual's life. See Supplementary Text S1 for the complete list of the religious experience scale items.
3. Religious *emphasis scale* (28). This scale reflects the extent to which parents emphasized religious behaviors during development. The scale consists of 10 items. The Cronbach's alpha for the God Emphasis Scale is 0.92. Examples of the items involved different religious behavior such as “*taking part in religious youth groups,” “praying before a meal,” or “Going to church: attending religious services*.” A higher score indicates a stronger emphasis parents gave to religious behaviors. See Supplementary Test S1 for the complete list of the religious emphasis scale.

An exploratory factor analysis (EFA) was conducted on the items of the God Image Inventory, Religious Experience Scale, and Religious Emphasis Scale. We then extracted the individualized factor scores for further use in subsequent analyses. The number of items per scale (10–14) and the sample size (*N* = 150) aligns with recent guidelines for exploratory factor analysis (34).

Specifically, we used the factor score regression method (35), standardizing the computed factor scores to a mean of zero and a standard deviation of one. This approach was chosen to enable standardized comparisons across scales.

#### 2.4.2 Attachment style measures

Assessment styles were measured using two different methods. The first method provided a categorical measure of attachment, based on participants' selection of the one attachment style that best described them [the Relationship Questionnaire (RQ); (24)]. The second method provided continuous scores for each attachment style by asking participants to rate themselves on four different attachment dimensions [the Relationship Scales Questionnaire (RSQ); (25)]. Both measures are widely used in the adult attachment literature (36).

1. The RQ (24) consists of a single-item measure, where participants were asked to identify their attachment style by reading four short paragraphs, each describing close relationships in adulthood, and selecting the one that best described them. In the second part, RQ2, participants are asked to rate their agreement with each prototype on a 7-point scale. The highest of the four attachment prototype ratings is then used to classify participants into an attachment category. The RQ was shown to be a valid tool in assessing attachment styles, in both healthy and clinical (37) samples.
2. The RSQ (25) is a 30-item self-report measure assessing four attachment styles: secure, fearful, preoccupied, and dismissive. Items are rated on a five-point scale, with mean scores calculated for each style. The measure has adequate reliability and convergent validity (25).

### 2.5 Statistical analyses

Prior to analyses, missing values (Religious emphasis scale, *n* = 5; God Image Inventory *n* = 10; Religious experience scale *n* = 9) were replaced by mean of the scale. Behavioral data analysis was carried out using JASP 0.19.3 (38) with the alpha level set to *P* < 0.05 (two-tailed).

First, given that we define CTG as a single construct measured repeatedly using three different tools, and that the assumption of homogeneity of variance was met (Levene's test, all *P* > 0.16), we used a repeated measures analysis of variance to evaluate the effect of attachment style on CTG measures. Effect size (η^2^) was calculated. Following recent recommendations for multiple statistical analyses to assess robustness (39), we complemented the frequentist approach with an equivalent Bayesian analysis. We report Bayes Factor (BF10) as an odds ratio, indicating the likelihood of the data under one hypothesis compared to another (40). A correlation analysis was used to evaluate the association between the level of endorsement of each attachment style (as measured by RSQ), with the three measures of connection to God. The assumption of data normality was not met (Shapiro-Wilk, *P* < 0.01), hence Spearman's Rho correlations were used.

## 3 Results

### 3.1 Associations between attachment style and measures of connection to God

#### 3.1.1 Secure attachment vs. non-secure attachment styles

The pTBI sample was split into a *secure group (*i.e., 57 individuals who endorsed a secure attachment style) and a *non-secure group* (i.e., 93 individuals who endorsed dismissing, fearful, or preoccupied style), based on responses to the categorical question from the RQ.

First, we conducted a repeated measures analysis of variance with the scores in the three measures of connection to God as repeated measures, and the attachment style (secure vs. non-secure) as a between-participant factor. We included age, years of education, and total brain volume loss (measured in cc) as covariates in this analysis.

**Figure 2:**
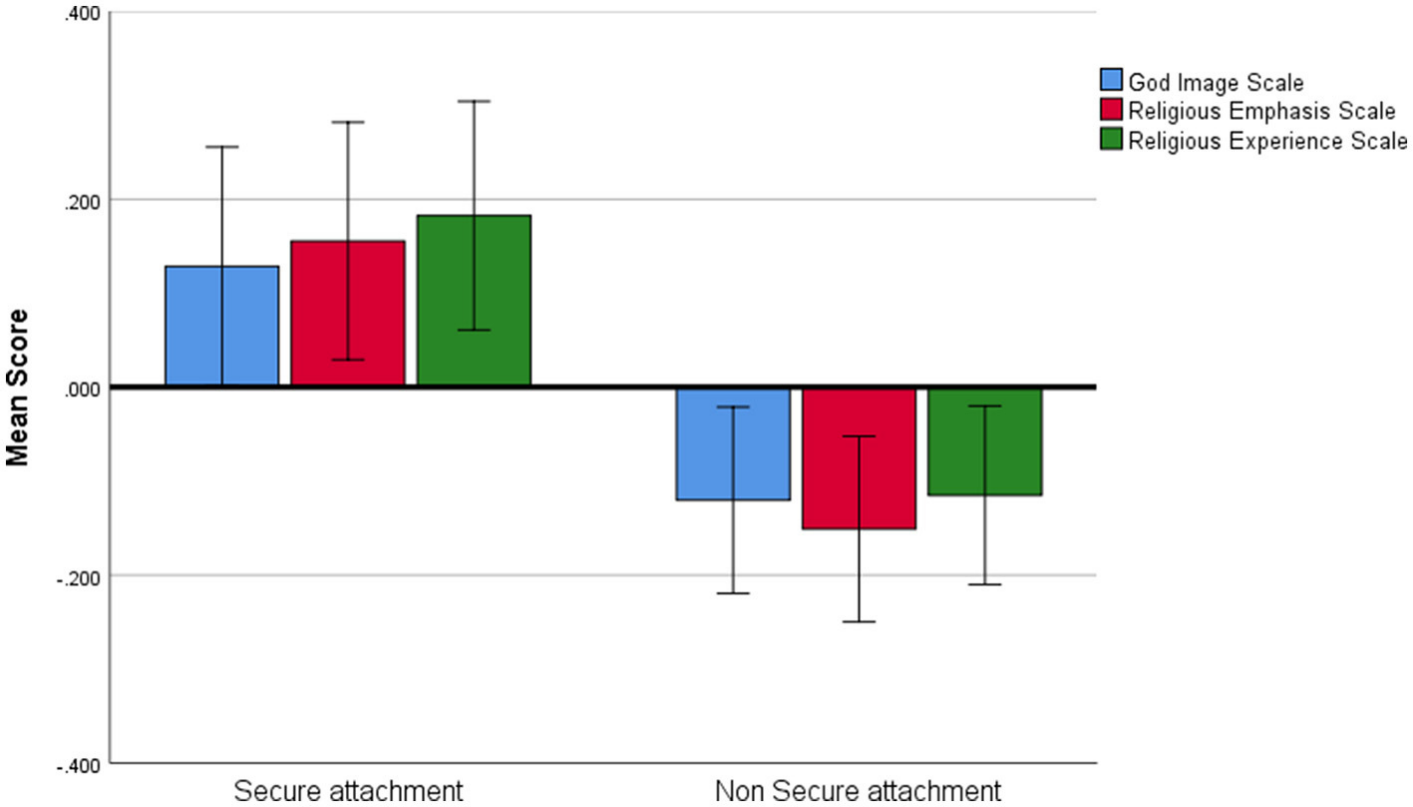
Measures of connection to God. Average scores on the three measures of connection to God in individuals with secure and non-secure attachment styles. Error bars represent one standard error.

We found a significant main effect for attachment style, such that individuals with secure attachment scored higher on the measures of connection to God compared to individuals with a non-secure attachment style (*F*_1,145_= 4.272, *P* = 0.041, η^2^ = 0.020; BF_10_= 0.618, Figure 2). There was no main effect of CTG measure nor was there interaction. A *post-hoc* analysis was subsequently conducted using the Bonferroni correction. No significant differences were found between secure and non-secure attachment styles on any individual CTG measure (all *P*_bonf_ > 0.5).

#### 3.1.2 Correlations between attachment styles and connection to God

Endorsements of each specific attachment style (as measured by RSQ) were correlated with the three measures of connection to God. A negative correlation was found between dismissing attachment style and all three measures of connection to God (see Table 2). This finding indicates that participants with a more dismissing orientation (avoiding intimacy, self-reliant) score lower on measures of CTG (report weaker connection to God and religious practice).

**Table 2:** Correlation matrix between attachment styles and measures of connection to God.

**Attachment style**		**Religious experience**	**God image**	**Religious emphasis**
Secure Attachment style	Spearman's rho	−0.035	−0.045	0.042
	*p*-value	0.678	0.6	0.621
Fearful Attachment style	Spearman's rho	−0.137	−0.065	−0.198
	*p*-value	0.106	0.447	0.017^*^
Preoccupied Attachment style	Spearman's rho	0.164	0.124	0.077
	*p*-value	0.052	0.145	0.357
Dismissing Attachment style	Spearman's rho	−0.294	−0.235	−0.228
	*p*-value	< 0.001^**^	0.005^**^	0.006^**^

Spearman's Rho correlations between each attachment style and measures of connection to God. ^*^p < 0.05; ^**^p < 0.01.

### 3.2 VLSM analysis

A whole-brain VLSM analysis was performed for pTBI participants with secure and non-secure attachment separately (grouping was made based on responses to the RQ). For each group, three analyses were conducted with the three connection to God measures as the outcome, and total brain volume loss as a covariate.

#### 3.2.1 VLSM results: secure attachment

**Figure 3:**
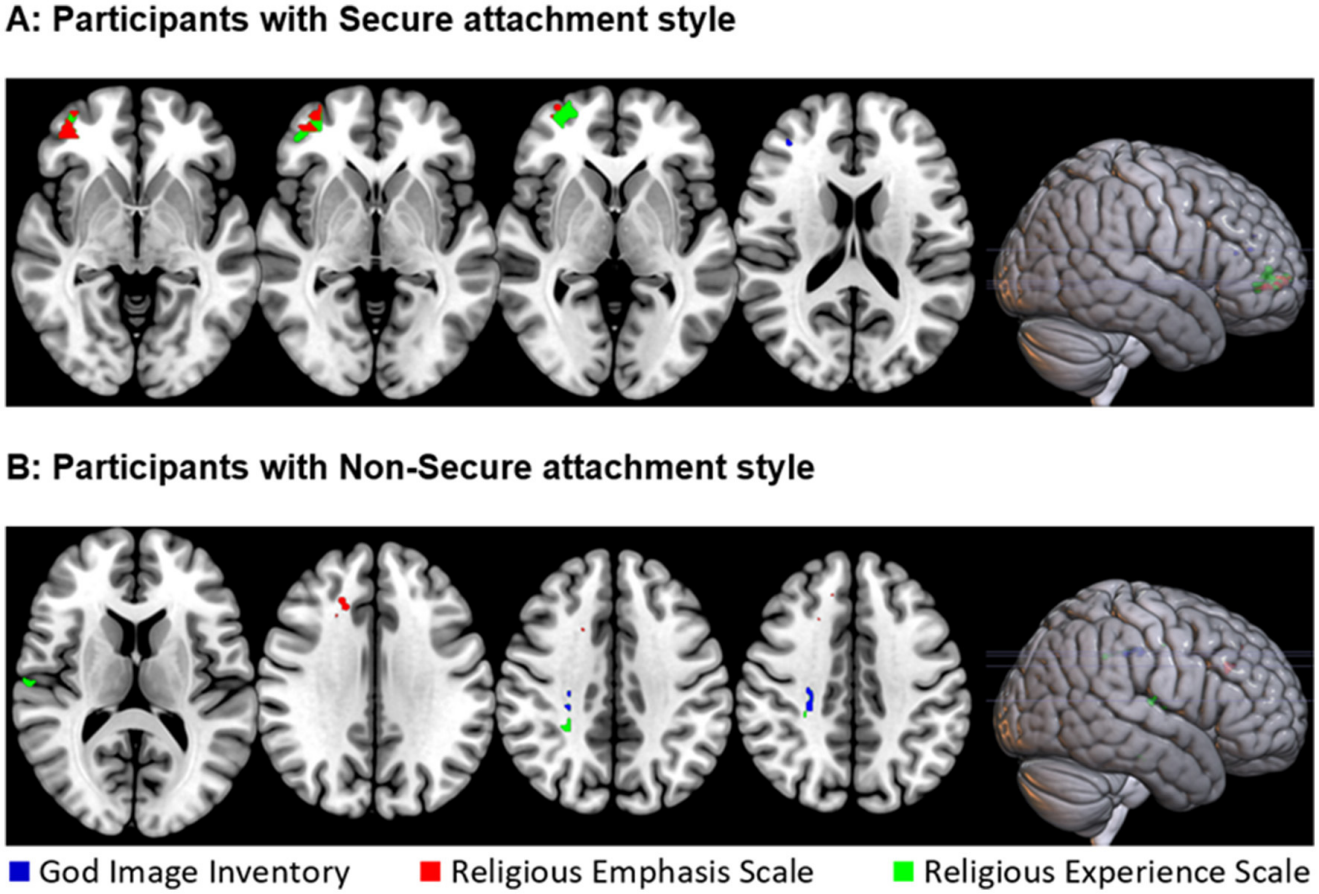
Location of brain lesions which significantly associates with stronger connection to God, as measured by the God image inventory (in blue), the religious emphasis scale (in red), and the religious experience (in green). All images are presented in radiological space (i.e., the right is shown on the left). **(A)** Results for participants with secure attachment. Z values (from left to right): −4, −2, 0, 18. **(B)** Results for participants with non-secure attachment. *Z* values (from left to right): 12, 32, 38, 40.

Whole-brain VLSM analysis on participants with secure attachment (*n* = 48) revealed two significant overlapping clusters. The first, for the religious emphasis scale (volume = 62 voxels, Max *t* = 1.76) was located predominantly in the orbital part of the middle and inferior frontal gyrus in the right hemisphere. The peak MNI coordinates were (34 44 −4). The second, for the religious experience scale (volume = 150 voxels, Max *t* = 2.48), cluster was located primarily within the orbital part of the inferior frontal gyrus in the right hemisphere. The peak MNI coordinates were (46 46−6). A smaller (volume = 3 voxels, Max *t* = 2.43) cluster was associated with the God image inventory, in the Middle frontal gyrus The peak MNI coordinates were (36 40 18; see Table 3 and Figure 3A).

**Table 3:** Voxel-based lesion-symptom analysis results.

**Outcome**	**Structure**	**Voxels**	**Peak MNI coordinates**			**Max *T*-Value**
			**x**	**y**	**z**	
**Religious emphasis**						
secure (*n* = 48)	Right middle and inferior frontal gyrus, orbital part	62	34	44	−4	1.76
Non-secure (*n* = 66)	Right cingulum	16	18	26	28	1.92
	Right superior frontal gyrus, medial	3	12	34	40	1.92
**Religious experience**						
secure (*n* = 45)	Right inferior frontal gyrus, orbital part	150	46	46	−6	2.48
Non-secure (*n* = 65)	Right corticospinal tract	34	26	−32	38	1.87
	Right superior temporal gyrus	14	58	−16	10	2.03
**God Image Inventory**						
Secure (*n* = 47)	Middle frontal gyrus	3	36	40	18	2.43
Non-secure (*n* = 63)	Right corticospinal tract	23	26	-32	38	1.95

Results from VLSM analyses showing regions of damage associated with higher scores on measures of connection to God, presented separately for the secure and non-secure groups. Regions were defined using the Automated Anatomical Labeling (AAL) atlas and the Natbrainlab atlas of white matter pathways.

#### 3.2.2 VLSM results: non-secure attachment

Whole-brain VLSM analysis on participants with an insecure attachment style (*n* = 66) revealed a few small clusters of < 50 voxels, mostly in white matter areas, including overlapping clusters for the religious experience and God Image scores at the right corticospinal tract. Other clusters were identified at the right cingulum, the right medial superior frontal gyrus, and the right superior temporal gyrus (see Table 3 and Figure 3B).

## 4 Discussion

This is the first study to investigate the neural correlates of connection to God in individuals with different attachment styles. Our findings suggest that attachment style can shape one's connection to God on the behavioral and the neuronal level, showing that (1) individuals with a secure attachment style report a stronger connection to God, and (2) the role of the right orbitofrontal cortex (OFC) in underlying CTG is different for individuals with secure and insecure attachment style. We will discuss the results and their implications, as well as the challenges in this area of research and future potential directions next.

### 4.1 Attachment style and connection to God: behavioral findings

Findings from this study suggest that individuals with secure attachment style form a stronger connection to God compared to individuals with an insecure attachment style, regardless of brain lesion location. These results are in line with previous studies reporting an association between secure attachment style and a closer connection to God or religious practice in the general population (41).

This finding supports the *correspondence hypothesis*, a concept in the psychology of religion that suggests that the nature of an individual's attachment style will correspond to their relationship with God. Specifically, if someone had a secure attachment with their caregivers, they are likely to perceive God as loving and caring, while those with insecure attachments might view God as distant or punitive (8, 42). However, it is important to note that the correspondence hypothesis is referring to *attachment* to God, while in this study we measured *Connection* to God which is a broader term.

We also found that dismissive attachment style is specifically associated with a weaker connection to God, regardless of brain injury. People with dismissing attachment style withdraw from intimate relationships and tend to be self-reliant. Our data is therefore in line with the expectation that people with dismissing attachment style will be more likely to face problems on their own and not reach out to God for support. Moreover, these results support previous findings reporting an association between dismissing attachment style and lack of intimacy in a relationship with God, in a sample of undergraduate college students (10). It is also in line with a study that showed that people with dismissing attachment style were less likely to plead to God compared to people with a preoccupied and secure attachment style (43).

### 4.2 Attachment style and connection to God: neuronal findings

Our findings indicate that the brain regions involved in an individual's CTG are influenced by their attachment style. For individuals with secure attachment, lesions to the right orbital part of inferior and middle frontal gyrus are associated with stronger connection to God, while for individuals with insecure attachment, those regions were not associated with a connection to God.

The observed link between the right-hemisphere connection to God aligns with previous research suggesting that the right hemisphere plays a dominant role in emotional processing and affective experiences (44–46), including those related to spirituality and religiosity (17, 47, 48).

#### 4.2.1 The orbitofrontal cortex and connection to God

The OFC is functionally related to the ventromedial prefrontal cortex (49), which is often associated with religious beliefs and a relationship with God (17, 21, 50). Moreover, the medial OFC uniquely is associated with subjectively rewarding and pleasant stimuli (51–53), which may include positive religious beliefs and experiences (54).

There are several potential explanations to the finding that individuals with a secure attachment style show a stronger connection to God after a lesion to the right OFC (rOFC). First, it is important to note that findings from this study do not necessarily imply that the rOFC directly impacts connection to God, even when it is intact. Instead, one possible interpretation is related to the rOFC role in modulating balanced emotional processing (55). It is possible that when the rOFC is damaged, individuals may experience reduced emotional stability. For those with a secure attachment style, a strong connection to God may serve as a compensatory source of emotional comfort, whereas individuals with an insecure attachment style may not find the same solace in their religious beliefs, and therefor do not show elevated connection to God following damage in this area.

Another possible interpretation is that the rOFC inhibits other intact brain areas that enable the strong relationship with God observed in the securely attached patients. This interpretation is challenged by the fact that regions that are known to share an antagonistic relationship with the OFC and the vmPFC [e.g., frontoparietal and lateral parietal regions (56, 57)] have shown to be involved in doubting religious belief and spiritual concepts (58). For instance, excitatory TMS to the right inferior parietal cortex reduced biases toward religious and spiritual concepts (47), and disbelief in the efficacy of prayer is associated with deactivations in the temporopolar/orbitofrontal regions (15). Yet, a different imaging study showed an association between activation in the right OFC to *increased* religious experience (48). Future research on the brain basis of connection to God is needed to identify areas directly regulated by the OFC, that when released from its inhibition allows for a stronger relationship with God, at least in securely attached patients.

Lastly, it is possible that given the role of the rOFC in psychological flexibility (59, 60) a damage in this area might result in stronger beliefs in religious fundamentalism and authoritarianism (61, 62).

### 4.3 Study limitations and future research direction

This study has a unique methodological strength which is achieved by combining behavioral data (attachment and connection to God) and neuroanatomical data (brain lesion mapping) in a large sample. Nevertheless, as with any study targeting a complex social concept, this study is not free of limitations which we acknowledge here.

First, we assessed attachment using brief, self-report measures. The assessment of attachment therefore reflects individuals' subjective perceptions of their close relationships, which may be vulnerable to reporting bias. Future research may benefit from adding interview methodologies over self-report assessments of adult attachment (63). Yet, it is important to note that the tools that were used in this study are among the most reliable of their kind, and are frequently used in research on adult attachment, providing consistent results (36).

Second, the sample in our study was composed of older male participants. Men and women may exhibit different attachment styles (64). Moreover, aging is associated with more important role for religion in one's life (65), and can influence cognitive and emotional changes. Therefore, the results may not be generalizable to younger or female samples and should be interpreted with these considerations in mind. However, it is important to note that age and gender cannot account for the observed differences between secure and insecure attachment groups reported in this study. Future studies are required to evaluate the generalizability of the results reported here on diverse samples of men and women, in different age groups and from different cultural backgrounds.

Lastly, it is important to note that the reported VLSM analysis did not include a correction for multiple comparisons, which fits the exploratory nature of the study. Correcting for multiple comparisons in this case could potentially obscure meaningful associations that may guide future research directions. Importantly, we were able to replicate our findings by conducting three separate VLSM analyses—one for each outcome measure—and obtaining similar results. Furthermore, the observed difference between secure and insecure attachment styles cannot be attributed to the lack of correction for multiple comparisons, as it remained consistent across all analyses. Nonetheless, future studies can focus on the rOFC a-priori as a region of interest and incorporate permutation analysis as correction (66, 67).

Although our study leaves some questions unresolved, it nevertheless offers new data that ties attachment style to connection to God using both behavioral and lesion mapping methods.

## 5 Conclusions

In conclusion, the present study provides evidence that attachment style impacts one's connection to God, with secure attachment being associated with stronger connection to God regardless of prior brain injury. It also suggests that attachment style impacts the brain regions associated with connection to God, with a lesion in the rOFC being associated with stronger connection to God in individuals with secure- and not insecure- attachment style. These findings deepen our understanding of how attachment style influences spiritual experiences, which can have implications for personalized approaches to spiritual care. Furthermore, this study enhances our understanding of the brain mechanisms underlying spiritual experiences, offering valuable insights into the neural substrates of faith and religiosity.

## Data Availability

The raw data supporting the conclusions of this article will be made available by the authors, without undue reservation.
