# Supplementary material for: Attachment style and its impact on connection to God in individuals with brain injury: behavioral and lesion-based findings
Source: Front Neurol. 2025 Apr 24;16:1488890. doi: 10.3389/fneur.2025.1488890 (PMC12058672; doi:10.3389/fneur.2025.1488890)
Supplement: Supplementary file 1 [file Data_Sheet_1.pdf]

Connection to God scales

[*] Inverted score

God Image Inventory (Lawrence, 1997)

1. God does not notice me. [*]
2. God lifts me up.
3. I am never really sure that God is really listening to me. [*]
4. God doesn’t feel very personal to me. [*]
5. I can talk to God on an intimate basis. [*]
6. I get no feeling of closeness to God, even in prayer. [*]
7. I feel that God knows me by name.
8. God never reached out to me. [*]
9. I feel warm inside when I pray.
10. God does not answer when I call. [*]
11. Prayer is very meaningful to me.
12. I prefer to face my problems without prayer. [*]
13. God tells me what he wants from me.
14. I do not think about God very often. [*]

Religious Emphasis Scale (Altemeyer, 1988)

1. Going to church: attending religious services
2. Attending “Sunday School”; getting systematic religious instruction regularly
3. Reviewing the teaching of the religion at home
4. Praying before meals
5. Reading Scripture or other religious materials
6. Praying before bedtime
7. Discussing moral “do’s” and “don’ts” in religious terms
8. Observing religious holidays; celebrating events like Christmas in a religious way
9. Being a good representative of the faith; acting the way a devout member of your religion would be expected to act
10. Taking part in religious youth groups

Religious Experience Questionnaire (Edwards, 1976)

1. I experience an awareness of God's love.
2. I pray privately in places other than church.
3. I experience feelings of anger or resentment toward God. [*]
4. I ask God to forgive my sins.
5. I am afraid that God is going to punish me in some way.
6. When I have decisions to make in my everyday life, I try to find out what God wants me to do.
7. I experience the felling that God is so big and important He doesn't have time for my personal problems. [*]
8. I feel very close to God in my prayer, during public worship, or at important moments in my life.
9. I experience awareness of God's influence in my daily life.
10. When I pray to God, I feel like I'm having a conversation with a close friend.
11. My relationship to God is characterized by close fellowship.
12. I find myself doubting that God really exists. [*]
